# Phylogeny and Biogeography of Hawkmoths (Lepidoptera: Sphingidae): Evidence from Five Nuclear Genes

**DOI:** 10.1371/journal.pone.0005719

**Published:** 2009-05-28

**Authors:** Akito Y. Kawahara, Andre A. Mignault, Jerome C. Regier, Ian J. Kitching, Charles Mitter

**Affiliations:** 1 Department of Entomology, College Park, Maryland, United States of America; 2 Center for Biosystems Research, University of Maryland Biotechnology Institute, College Park, Maryland, United States of America; 3 Department of Entomology, The Natural History Museum, London, United Kingdom; Max Planck Institute for Evolutionary Anthropology, Germany

## Abstract

**Background:**

The 1400 species of hawkmoths (Lepidoptera: Sphingidae) comprise one of most conspicuous and well-studied groups of insects, and provide model systems for diverse biological disciplines. However, a robust phylogenetic framework for the family is currently lacking. Morphology is unable to confidently determine relationships among most groups. As a major step toward understanding relationships of this model group, we have undertaken the first large-scale molecular phylogenetic analysis of hawkmoths representing all subfamilies, tribes and subtribes.

**Methodology/Principal Findings:**

The data set consisted of 131 sphingid species and 6793 bp of sequence from five protein-coding nuclear genes. Maximum likelihood and parsimony analyses provided strong support for more than two-thirds of all nodes, including strong signal for or against nearly all of the fifteen current subfamily, tribal and sub-tribal groupings. Monophyly was strongly supported for some of these, including Macroglossinae, Sphinginae, Acherontiini, Ambulycini, Philampelini, Choerocampina, and Hemarina. Other groupings proved para- or polyphyletic, and will need significant redefinition; these include Smerinthinae, Smerinthini, Sphingini, Sphingulini, Dilophonotini, Dilophonotina, Macroglossini, and Macroglossina. The basal divergence, strongly supported, is between Macroglossinae and Smerinthinae+Sphinginae. All genes contribute significantly to the signal from the combined data set, and there is little conflict between genes. Ancestral state reconstruction reveals multiple separate origins of New World and Old World radiations.

**Conclusions/Significance:**

Our study provides the first comprehensive phylogeny of one of the most conspicuous and well-studied insects. The molecular phylogeny challenges current concepts of Sphingidae based on morphology, and provides a foundation for a new classification. While there are multiple independent origins of New World and Old World radiations, we conclude that broad-scale geographic distribution in hawkmoths is more phylogenetically conserved than previously postulated.

## Introduction

The hawkmoths (Lepidoptera: Sphingidae), comprising about 1400 species in some 200 genera and found on every continent except Antarctica [1], [2], are one of the most conspicuous and well-studied insects. Due in part to their large size, sphingids have long provided models for studies of genetics, physiology and development [3]–[8], functional morphology [9], [10], plant-herbivore interactions [e.g.], [ 11], [12]–[14], pollination biology [e.g.], [ 15], [16]–[21], and biogeography [22]. Sphingids are also a focal group for biodiversity and habitat quality assessment studies [23]. Some species are agricultural pests [24], [25], while others have been used as biological control agents [26]. Recently, sphingids have also become a model group for testing the reliability of DNA barcoding for species identification [27], [28].

Research of many kinds on hawkmoths would benefit from a more robust phylogenetic/comparative framework for this family than is currently available. Morphological and molecular analyses strongly support the monophyly of Sphingidae, and placement within the superfamily Bombycoidea [2], [29]–[31]. Within the family, however, there has yet to be a comprehensive study of relationships based on explicit phylogenetic methodology. A preliminary molecular analysis by Regier et al. [32], while spanning all three subfamilies, included just fourteen species. All other analyses have focused within a tribe or genus (e.g., Acherontiini [33], [34], *Hyles* [35]–[37], *Proserpinus* [38]).

**Figure 1:**
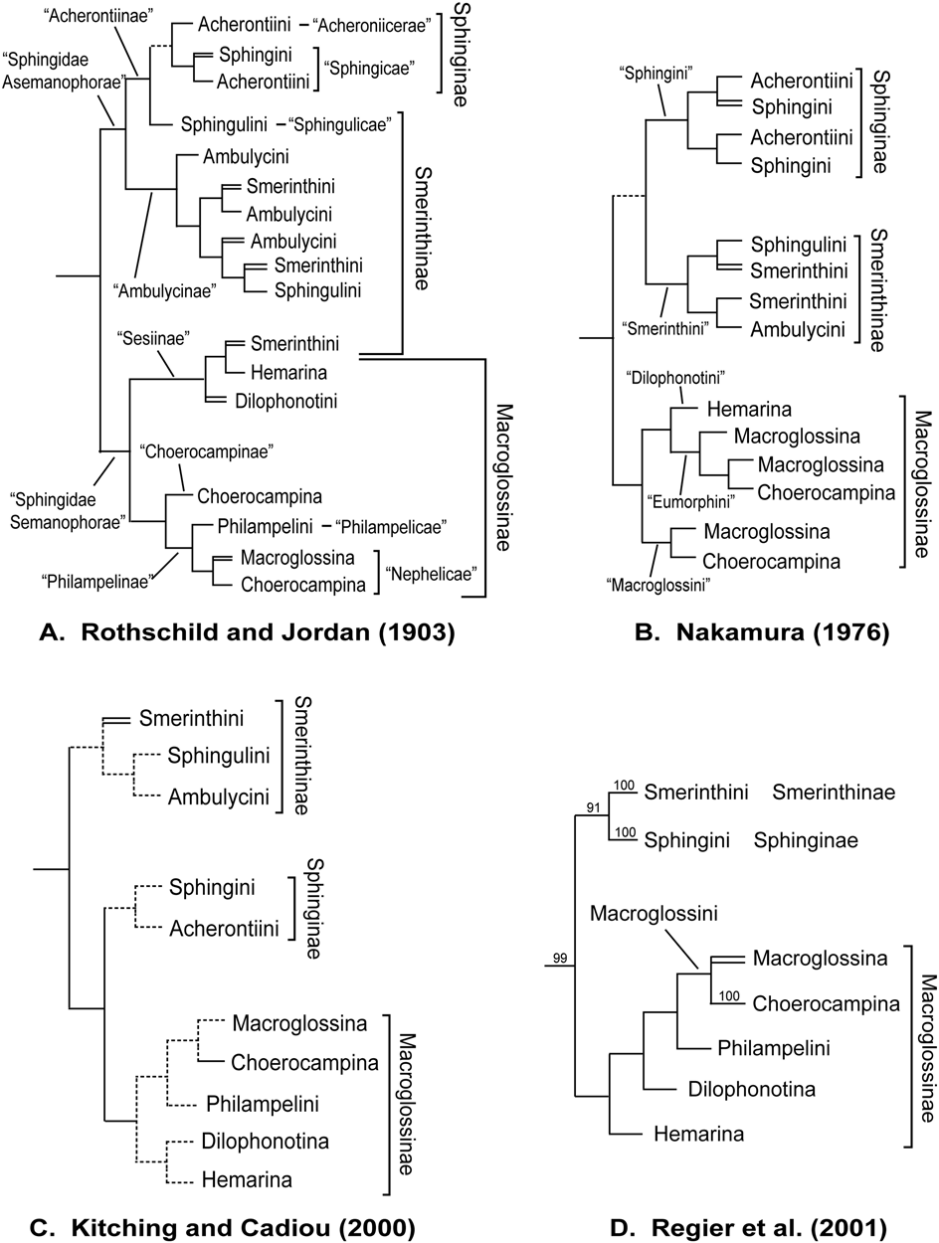
Hypothesized relationships of Sphingidae based on: A. Adult morphology [1], B. Larval and pupal morphology [44], C. Larval, pupal, and adult morphology [2], D. Molecular data (*EF-1α* and *DDC*) [32]. To facilitate comparison, all the trees have been reduced to show only the dispositions of the subfamilies, tribes and subtribes recognized by Kitching and Cadiou [2]. Nomenclature of previous authors, where different, is shown in quotes. A double line leading to a taxon name denotes paraphyly, a dotted line indicates uncertain relationships. Numbers above branches in Figure 1D are bootstrap values.

The present study builds on past and ongoing efforts to reconstruct sphingid phylogeny using morphology (Figs. 1A–C). In their monumental revision, the starting point for subsequent classifications, Rothschild and Jordan [1] divided the hawkmoths into two “series,” “Sphingidae Semanophorae” and the “Sphingidae Asemanophorae,” distinguished by the presence or absence of a patch of short sensory hairs (microtrichia) on the inner surface of the first segment of the labial palp. These groups correspond approximately to the present-day Macroglossinae and (Smerinthinae+Sphinginae) respectively (Fig. 1A). Writing long before the formalization of modern cladistics [39]–[41], Rothschild and Jordan presented a “tree” on which all then-known sphingid genera were placed according to shared derived morphological structures, although they circumscribed some taxonomic groups on the basis of symplesiomorphic resemblance.

Within their two series, Rothschild and Jordan recognized five subfamilies, all of which correspond approximately to modern groups, although many names and ranks have changed (Fig. 1A). Janse [42] reduced Rothschild and Jordan's subfamilies to tribes, and treated Semanophorae and Asemanophorae as subfamilies (Semanophorinae and Asemanophorinae), names that Hodges [43] changed to Macroglossinae and Sphinginae, based on the type genus of each subfamily. Minet [29] separated Sphinginae *sensu lato* into Smerinthinae and Sphinginae *sensu stricto*, in part because he believed that Sphinginae *sensu lato*, defined by the absence of the microtrichial patch, might be paraphyletic.

The next major contribution to sphingid systematics came from Nakamura [44]–[46], who reclassified several sphingid tribes and proposed relationships among Japanese hawkmoth genera based on characters of the larva and pupa (Fig. 1B), although without explicit cladistic analysis. He considered Smerinthini and Sphingini to be sister groups. His remaining tribes formed a group that roughly corresponds to Macroglossinae as defined in the most recent comprehensive revision of sphingid classification, that of Kitching and Cadiou [2]. In addition to stabilizing sphingid taxonomy and revising the tribal and subtribal level classification, Kitching and Cadiou proposed provisional relationships among genera (Fig. 1C), based in part on unpublished morphological analyses. Unlike earlier authors, they tentatively placed the Smerinthinae at the base of the family, postulating that Sphinginae plus Macroglossinae constitute a monophyletic group. Within Smerinthinae, Smerinthini was hypothesized to be paraphyletic. While putative apomorphies were identified for each subfamily [29], Kitching and Cadiou expressed caution about the monophyly of most tribes and subtribes, particularly within Smerinthinae.

The molecular analysis by Regier et al. [32], based on the nuclear genes elongation factor-1α (*EF-1α*) [47] and dopa-decarboxylase (*DDC*) [48], provided an initial test of Kitching and Cadiou's classification. Overall, their results (Fig. 1D) appeared to favor the conclusions of Rothschild and Jordan [1] and Nakamura [44], as Smerinthinae were grouped with Sphinginae to the exclusion of Macroglossinae. Further evidence was needed, however, as the number of genera was small and three key tribes, Acherontiini, Ambulycini, and Sphingulini, were not included.

In this report we expand gene sampling of Regier et al. [32] to five protein-coding nuclear genes, and taxon sampling to 131 exemplars representing 106 sphingid genera. We use these data to test the monophyly of, and to estimate phylogenetic relationships within and among, all of the subfamilies, tribes and subtribes in the classification of Kitching and Cadiou [2]. We then provide a preliminary assessment of the new phylogeny's utility for understanding sphingid biogeography. We investigate the scale, if any, on which distribution is phylogenetically conserved, allowing inference about the geographic setting of major events in hawkmoth evolution.

## Materials and Methods

### Taxon sampling

We sequenced all sphingid genera for which we were able to obtain alcohol-preserved material. We included one additional taxon (*Kloneus babayaga*) for which a nucleic acid extract was kindly provided by Mehrdad Hajibabaei of the Canadian Centre for DNA Barcoding at University of Guelph. Sequences for several taxa were obtained from previously published studies [31], [32], [49], [50]. In total, 131 sphingid species in 106 genera were included, and they are listed in Table S1. All generic names follow the classification of Kitching and Cadiou [2] except *Lintneria*, which was reinstated for 21 largely Neotropical species of *Sphinx* [51], and *Arctonotus*, which was recently synonymized with *Proserpinus* [52]. As outgroups we included ten exemplars sequenced by Regier et al. [53] that represent eight other families placed in the Bombycoidea *sensu stricto* of Minet [29], [54] and Lemaire and Minet [30].

### Gene sampling

Characters for this study come from five nuclear gene regions, totaling 6793 bp, which have previously proven useful for lepidopteran phylogenetics [55]. These include 2929 bp of *CAD* [56], 1282 bp of *DDC* [48], 1228 bp of *EF-1α* [47], 951 bp of *period* [57], and 403 bp of *wingless* [58]. GenBank accession numbers are listed in Table S1 and the entire aligned data matrix is available as supporting information (Dataset S1).

### Nucleic acid extraction, RT-PCR, and primer sequences

Our nucleic acid extractions, mostly from adult moths, were generally taken from the head or prothorax, though in a few cases a leg was used because the rest of the body was unavailable (e.g., *Aleuron chloroptera*, *Deidamia inscriptum*). Nucleic acid extractions were conducted with the Promega SV Total RNA Isolation System [59], with slight protocol modifications (exclusion of part IV. E. steps 4, 5) to permit extraction of both genomic DNA and RNA. All specimens and extractions are stored at −85°C in the AToLep Collection at the University of Maryland. Wing voucher images of specimens sequenced can be viewed at the LepTree website (www.leptree.net).

Selective amplification of gene coding regions was conducted using the reverse transcription polymerase chain reaction (RT-PCR) to avoid introns, and because RT-PCR yielded better results than DNA-PCR in previous experiments [55]. All primer sequences are bipartite [60], containing a specific, 18 nt, M13 sequence at the 5′ end, and a gene-specific, 17–32 nt, degenerate sequence at the 3′ end. Protocols and strategies used for RT-PCR amplification for each gene are available online [55].

### Data matrix construction

Sequence chromatograms were checked for accuracy and contigs edited and assembled with the Staden GAP4 software package [61]. Sequence alignment for each locus was conducted with MAFFT 6.611 [62] and manually checked with the Genetic Data Environment (GDE) software [63]. Each matrix was converted from FASTA to NEXUS format [64] and sequentially combined to create a single concatenated matrix of five genes using the “New Matrix Merge” command in WinClada [65].

### Phylogenetic analysis

An optimal model for nucleotide maximum likelihood (ML) inference was chosen under the Akaike Information Criterion (AIC) [66] from 56 different models as implemented in Modeltest 3.7 [67]. In all cases, the best model was determined to be the general-time-reversible substitution model [68], [69], with among-site-rate-heterogeneity modeled according to a gamma distribution (Γ) [70] while allowing for a proportion of invariable sites (I) [71]. ML analyses were conducted using the multi-threaded GARLI 0.96b8 [72]. Searches began with a random starting tree, and none of the default settings was changed except for the number of generations to termination, which was increased to improve the search for the optimal solution (genthreshfortopoterm = 20,000). To enhance further the search for best tree, the same search process was repeated 1000 times with a different starting seed, and the best tree was chosen. Optimal tree searches were executed in parallel using Grid computing [73] through The Lattice Project [74]. A GARLI Grid service was written using GSBL, a high-level Application Programming Interface (API) for writing Globus-based Grid services [75]. Non-parametric ML bootstrap analyses were also conducted on the Grid, each consisting of 2000 bootstrap replicates with default settings.

Maximum parsimony analyses (MP) were conducted with WinClada [65] and NONA [76]. Heuristic searches were computed with the following commands: hold 1000, hold/100, mult*100, max*. Congruence between multiple most-parsimonious cladograms (MPCs) was assessed with a strict consensus [77]. Branch support was assessed with Bremer support (BS) [78], [79] and the bootstrap [80]. Bremer support values were calculated in TreeRot 2c [81], and subsequent command files executed in PAUP*4b10 [82]. MP bootstrap values were computed in NONA [76] with 500 replications, 100 search replications (mult*100), and holding 10 starting trees (hold/10). Throughout this report, for the sake of consistency, we adopt the somewhat arbitrary conventions that “moderate” node support means bootstrap values of 70–79%, “strong” support means values of 80–89%, and “very strong” support means values of 90% and above.

To evaluate the relative contribution of each gene to the total phylogenetic signal, and to assess conflicts among genes, ML bootstraps were calculated separately for each of the one-gene data sets. As a complementary approach, we also carried out “partition addition bootstrap alteration”, or PABA [83], on a series of four-gene data sets obtained by removing each of the genes in turn. NEXUS format files of all combinations of the remaining four genes were constructed with the same methods as the combined five-gene data set.

### Biogeography

To explore broad biogeographic patterns in hawkmoths, we first determined, from various sources [e.g.], [43], [84,85], the distribution of each exemplar species across the traditional global biogeographic regions [86], [87]. We then simplified these distributions into a two-state character contrasting Old and New World, to test Kitching and Cadiou's [2] postulate of an Old World ancestral origin for the Sphingidae. We used Mesquite 2.5 [88] to calculate ancestral state conditions for biogeography in both an ML and MP framework. The Mkv model of Lewis [89], using the default search strategy of Mesquite, was chosen for all ML ancestral state reconstructions. Branch lengths were estimated from the molecular phylogeny.

## Results

### Phylogenetic analyses of five concatenated genes

**Figure 2:**
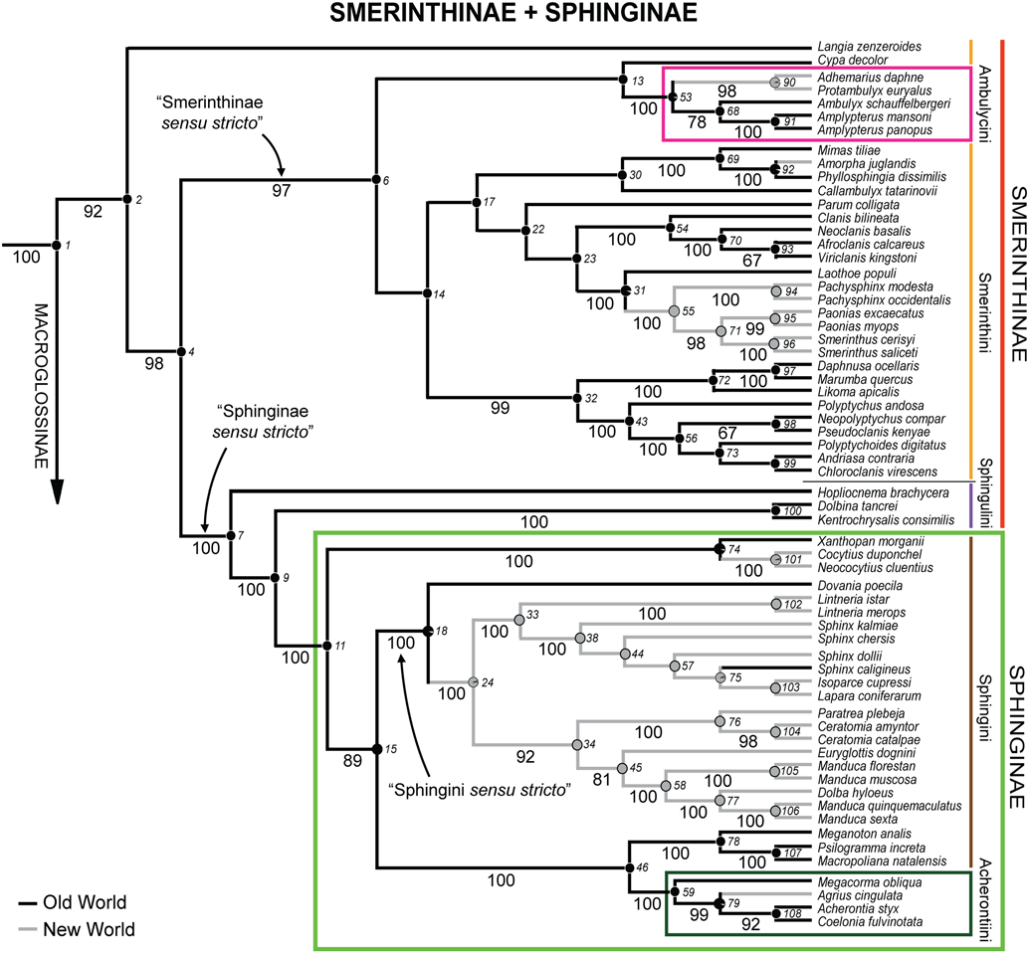
ML tree from combined five-gene simultaneous analysis, Smerinthinae+Sphinginae clade. The classification used here follows Kitching and Cadiou [2]. Larger-font numbers below branches are bootstrap values (>50%), smaller-font numbers to right of nodes are node numbers. Pie diagrams on nodes show proportions of total ancestral biogeographic state likelihood contributed by the states Old World (black) versus New World (gray), under ML (MK1).

**Figure 3:**
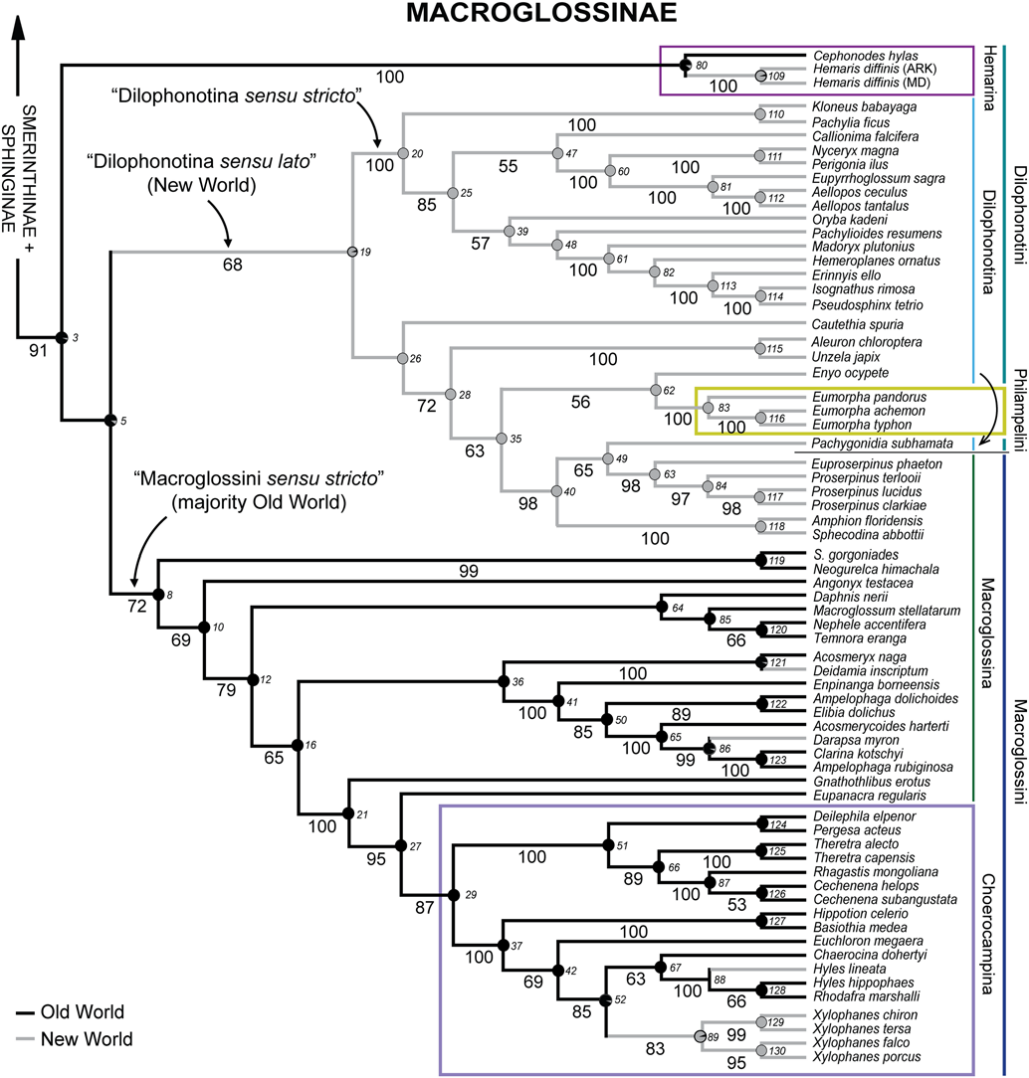
ML tree from combined five-gene simultaneous analysis, Macroglossinae clade.

The ML tree for the five genes combined, with associated bootstrap (BP) values, is shown in Fig. 2 and 3, while the corresponding MP tree is shown in Figure S1. The two trees were similar in topology, fully resolved or nearly so, and showed strong bootstrap support (≥80%) for more than two-thirds of the possible nodes. However, MP was susceptible to long-branch attraction (see discussion), and for this reason, the exposition below focuses on the ML tree except as otherwise noted. Relationships among outgroups, not shown here, were identical to those reported by Regier et al. [53].

The ML tree showed strong signal either for or against nearly all of the fifteen subfamily, tribal and sub-tribal entities recognized by Kitching and Cadiou [2], as well as many other groupings within and among these postulated by those authors (see also Table 1). Thus, our data set provides a strong test of those authors' hypothesis of classification and phylogeny. A detailed comparison of the present results to the hypothesis of Kitching and Cadiou [2] is provided in the Discussion.

**Table 1:** Recovery and bootstrap support for 20 selected clades under ML analysis.

Taxon	Node	ALL	CAD	DDC	EF-1α	PER	WG	-CAD	-DDC	-EF-1α	-PER	-WG
Sphingidae	1	**100**	**96**	**95**	54	*	*	**60 (40)**	99 (1)	100 (0)	100 (0)	99 (1)
Smerinthinae+Sphinginae	2	**92**	75	64	*	*	*	**64 (28)**	84 (8)	86 (6)	92 (0)	92 (0)
Smerinthinae+Sphinginae^1^	4	**98**	**94**	64	*	*	*	**73 (25)**	98 (0)	95 (3)	96 (2)	97 (1)
Macroglossinae	3	**91**	79	69	*	*	*	**49 (42)**	88 (3)	92 (−1)	93 (−2)	90 (0)
Sphingulini+Sphinginae	7	**100**	**100**	**99**	*	70	*	98 (2)	100 (0)	100 (0)	100 (0)	100 (0)
Sphinginae	11	**100**	**100**	76	*	54	**83**	98 (2)	100 (0)	100 (0)	100 (0)	100 (0)
Sphinginae^2^	15	**89**	*	**85**	*	68	*	95 (−6)	**45 (44)**	85 (4)	**60 (29)**	88 (1)
Dilophonotina *sensu stricto*	20	**100**	**98**	**99**	*	73	*	96 (4)	99 (1)	100 (0)	100 (0)	100 (0)
*C+E+G*	21	**100**	*	**99**	*	*	*	99 (1)	99 (1)	100 (0)	100 (0)	100 (0)
*C*+*E*	27	**95**	*	*	*	*	*	95 (0)	98 (−3)	93 (2)	**58 (37)**	**85 (10)**
Choerocampina	29	**88**	65	*	*	*	*	94 (−6)	**73 (15)**	**61 (27)**	**44 (44)**	**76 (12)**
*Sphinx* group	33	**100**	**100**	**100**	**95**	**99**	**90**	100 (0)	100 (0)	100 (0)	100 (0)	100 (0)
*Polyptychus* group	43	**100**	**100**	**89**	73	**84**	52	96 (4)	100 (0)	100 (0)	100 (0)	100 (0)
Ambulycini	53	**100**	**100**	**100**	*	**96**	*	98 (2)	100 (0)	100 (0)	100 (0)	100 (0)
*Clanis* group	54	**100**	**100**	**97**	62	55	*	99 (1)	100 (0)	100 (0)	100 (0)	100 (0)
Acherontiini	59	**100**	**100**	**94**	75	63	*	99 (1)	100 (0)	100 (0)	100 (0)	100 (0)
*Proserpinus* group	63	**98**	**99**	**100**	**95**	**99**	**90**	99 (−1)	98 (0)	100 (−2)	98 (0)	97 (1)
*Marumba* group	72	**100**	**98**	**94**	**96**	69	*	99 (1)	100 (0)	100 (0)	100 (0)	100 (0)
Hemarina	80	**100**	**100**	**100**	**98**	**98**	**85**	100 (0)	100 (0)	100 (0)	100 (0)	100 (0)
Philampelini	83	**100**	-	**100**	**100**	-	-	100 (0)	99 (1)	100 (0)	100 (0)	100 (0)

**Legend**: Results for five genes combined (‘ALL’), individual genes (next five columns), and all possible combinations of four genes with one excluded (denoted by minus signs in the last five columns). Numbers in parentheses are differences in BP value from the five-gene analysis. BP values≥80% (one and five-gene analyses) and differences ≥10% (four-gene analyses) are in bold. Asterisks denote clades that have BP<50%; hyphens mark clades absent from the analysis because of incomplete taxon sampling. *C* = Choerocampina, *E* = *Enpinanga*, *G* = *Gnathothlibus*.

1 Excluding *Langia*.

2 Excluding *Cocytius* group.

### Contributions of, and conflict among, individual genes

To characterize the individual gene contributions (or lack thereof) to the overall phylogenetic signal, we focused on a set of twenty relatively deep divergences (Table 1) that were strongly supported by the combined data (all BP≥88%; 16/20 with BP≥95%). When each gene was analyzed independently, all made strong contributions to at least some of these nodes (Table 1). The gene with the longest sequence, *CAD*, provided strong support (BP≥80%) for 13 of those nodes when analyzed independently. *DDC* alone strongly supported 14 nodes, despite a sequence length less than half that of *CAD*. *EF-1α* strongly supported five of the 20 nodes, *period* five, and *wingless* four. Most nodes were strongly supported by one or more individual genes, but four (nodes 2, 3, 27 and 29) had high combined-gene BP despite lack of strong support from any single gene, suggesting that additivity of weak but compatible signal from multiple genes accounts for about a fifth of these twenty instances of strong overall resolution. No gene strongly supported relationships that conflicted with those found in the all-gene tree, suggesting a general lack of marked conflict among genes.

The four-gene analyses provided a complementary assessment of individual gene contributions and conflict (Table 1). A decrease in BP for a node when a given gene is excluded, as compared to the all-genes analysis, was evidence that the gene provided support for that node. An increase in BP, conversely, implied that the gene conflicted with the node. We adopted the somewhat arbitrary heuristic criterion that a ≥10% change in BP represents a “significant” difference. Results from the four-gene analyses mirrored those from the single-gene analyses. Exclusion of *CAD* resulted in decreased BP support for thirteen nodes, four of which showed highly “significant” differences (≥25% BP). For the remaining genes, the corresponding numbers were: *DDC*, eight decreases, two “significant”; *EF-1α*, five decreases, one “significant;” *period*, four decreases, three “significant”; and *wingless*, six decreases, two “significant.” There was at least one apparent conflict (increase in BP upon exclusion) for each gene except wingless (*CAD*: 3; *DDC*: 1; *EF-1α*: 2; *period*: 1). However, these differences were mostly less than 5%, and all less than 10%, providing further evidence against strong conflict among genes.

### Biogeography

On a broad, intercontinental scale, geographic distribution appeared to be strongly conserved on the ML phylogeny (Figs. 2, 3). Ancestral distribution can be confidently inferred for most clades at all levels. Thus, as we have seen, Smerinthinae+Sphinginae comprise an ancestrally and still predominantly Old World lineage, giving rise to substantial secondary New World radiations within Ambulycini, Smerinthini and Sphingini. Within these larger clades restricted mainly to one hemisphere are occasional inferred dispersals to the other. Examples include apparent dispersals to the Nearctic by ancestral *Amorpha*, to the Palearctic within *Sphinx*, and to the Neotropics within the *Cocytius* group and *Agrius*. Within Macroglossinae, there is a strong dichotomy between the clades comprising the predominantly Old World Macroglossini *sensu stricto* and New World Dilophonotina *sensu lato*, but there also appear to have been recent dispersals back to the New World in at least four nested lineages within Choerocampina and Macroglossina. Parsimony and likelihood also assigned an Old World ancestor to Macroglossinae (Fig. 3), and to the family as a whole, but this inference is somewhat less secure given our limited taxon sampling (see Discussion).

## Discussion

### Monophyly of Sphingidae, basal divergences, and subfamily definitions/relationships

Our analysis strongly corroborates morphological evidence for the monophyly of the Sphingidae [1], [2], [29], and supports a basal divergence within the family between Smerinthinae+Sphinginae (BP = 92%) and Macroglossinae (BP = 91%). Overall, the Smerinthinae+Sphinginae lineage most closely matches the morphological trees of Rothschild and Jordan [1], and Nakamura [44], but as yet no definitive morphological synapomorphy is known. While not yet objectively tested using morphology, monophyly of Macroglossinae was previously hypothesized on the basis of labial palp and pupal characters [2], [44]. Support for the monophyly of Macroglossinae came predominantly from *CAD*, as it provided the highest bootstrap support when analyzed alone, and bootstrap dropped from 91% to 49% when it was excluded (Table 1). *CAD* constitutes nearly 45% of the data set, so its major contribution to the node in question is expected. Monophyly of Smerinthinae+Sphinginae, and also for the Macroglossinae, however, was substantially lower in the MP analysis. Examination of the individual MP bootstrap trees revealed several long-branched taxa that were frequently grouping with outgroups and lowering bootstrap support (see below for further discussion on long-branch attraction). Basal divergences within Sphingidae are congruent with those of the much smaller molecular study by Regier et al. [32].

Within the Smerinthinae+Sphinginae lineage, our tree renders both Smerinthinae and Smerinthini paraphyletic with respect to Sphinginae by favoring very strongly a basal split between *Langia zenzeroides* and all remaining taxa (node 4, BP = 98%, Fig. 2). The position of *Langia* is unique in that it may illustrate the plesiomorphic condition of the Smerinthinae+Sphinginae. While an explicit analysis of optimization is necessary, *Langia*, Sphingulini, and most Smerinthinae share a larva that has a pointed triangular head, a full set of oblique lateral stripes, a rough “shagreened” skin, and an adult with a non-functional tongue.

Monophyly of Smerinthinae *sensu lato*, Smerinthini, and Sphingulini had previously been doubted on multiple morphological grounds (e.g., possession by various subgroups of Smerinthini of features found in other hawkmoths [1], [2], [44]). Smerinthinae *sensu lato* seem clearly paraphyletic, but our data nonetheless very strongly support monophyly for the majority of this subfamily, consisting of Ambulycini and Smerinthini minus *Langia*. Sphingulini is also paraphyletic, with *Dolbina*+*Kentrochrysalis*, to the exclusion of an Australian group represented here by *Hopliocnema*, comprising the sister group to Sphinginae (node 11, BP = 100%). We make no formal nomenclatural proposals here, but it would be reasonable to treat Sphinginae+Sphingulini as Sphinginae *sensu stricto* (node 7, BP = 100%), and its sister group, Ambulycini and Smerinthini minus *Langia*, as Smerinthinae *sensu stricto* (node 6, BP = 97%).

### Relationships within “Smerinthinae sensu stricto” (Ambulycini+Smerinthini [minus *Langia*])

Monophyly of Ambulycini is strongly supported by molecules (node 53, Fig. 2) and by morphology (I. J. Kitching, unpublished data, 1993). All pupae examined so far in this tribe have a unique notch at the base of the cremaster [45] and many species also typically share larval host plants in Anacardiaceae or Lauraceae [90]. The tribe as sampled here divides into Neotropical (*Adhemarius*+*Protambulyx*) and Old World (*Ambulyx*+*Amplypterus*) sister groups. Kitching and Cadiou [2] hypothesized, however, that some Old World Ambulycini not sampled here (e.g., *Akbesia*, *Batocnema*, *Compsulyx*) may be more closely allied to the Neotropical species than to *Ambulyx*+*Amplypterus*, based on similarities of the hindwing eyespot pattern and a spinose gnathos in the male genitalia. These relationships would reinforce the inference of an Old World ancestor for Ambulycini.

Smerinthini, even excluding *Langia* and Sphingulini, are a morphologically heterogeneous group for which morphological synapomorphies are lacking. Basal divergences in this assemblage are weakly supported by molecular data, and monophyly can be neither confirmed nor confidently rejected. However, the molecular analysis provides strong support for a number of subgroups previously identified, at least in part, on morphological grounds [2]. Thus, monophyly is very strongly supported for both the *Marumba* group (node 72) and the *Polyptychus* group (with the addition of *Chloroclanis*, node 43) of Kitching and Cadiou [2], as well as a sister group relationship between these. Together these form a very strongly supported predominantly Afrotropical clade (node 32) with an apparent behavioral synapomorphy: in all species for which the immature stages are known, the first instar larva consumes only the eggshell before molting into the second instar [84], [91], [92]. Strong support is also found for another predominantly African clade, identified in part by Kitching and Cadiou [2], that is centered on *Clanis* (node 54).

*Laothoe*, *Pachysphinx*, *Paonias*, and *Smerinthus* form a very strongly supported Holarctic clade (node 31), identified in part by Kitching and Cadiou [2], within which generic relationships are strongly resolved. *Laothoe*, distributed from Ireland to Japan, feeds on *Populus* and *Salix* [84], which are also the sole larval hosts of the Nearctic *Pachysphinx* [90]. In contrast, one of the two most-recently diverging genera, *Paonias*, is polyphagous, feeding on a variety of host plants that differ in chemical composition [90]. Relationships among these genera suggest a single invasion from Old World to New World, followed by development of the full adult hindwing eyespot in the Nearctic *Paonias* and *Smerinthus*, a shift from monophagy to polyphagy in the former genus and reinvasion of the Palearctic by one or more lineages of the latter. Finally, the molecular data strongly corroborate the predicted sister group relationship between the eastern Nearctic *Amorpha* and the eastern Palearctic *Phyllosphingia*, both restricted to Juglandaceae [2], as well as a close relationship of these to the Palearctic *Mimas*.

### Relationships within Sphinginae: Acherontiini and Sphingini

Basal divergences within Sphinginae are strongly resolved in our analyses (BP≥89%, Fig. 2). The first lineage to branch off comprises the Palaeotropical genus *Xanthopan* plus the Neotropical *Cocytius/Neococytius* (node 74, BP = 100%). This grouping, previously suggested by the shared possession of extremely long tongues [93], had been questioned on other morphological grounds [33], [34]. The remaining sphingines are split, with 100% BP support, into a larger, mostly New World clade containing the great majority of Sphingini (node 18), and a smaller, Old World clade consisting of the Acherontiini and their nearest relatives among the Sphingini (node 46). Monophyly of Acherontiini, strongly corroborated here, is supported by a labial palp feature and three characters of the genitalia [33], [34]. Relationships among the acherontiine genera included here are congruent with Kitching's expanded morphological parsimony analyses of this tribe [33], [34]. The sister clade to the Acherontiini is a set of genera centered on *Psilogramma* (node 78, BP = 100%), phylogenetic proximity among which had been previously predicted [2].

The phylogenetic placement of Acherontiini within Sphingini renders the latter paraphyletic. One reasonable way to achieve monophyly for tribes of Sphinginae would be to confer tribal status separately on the *Psilogramma* group, the *Xanthopan*/*Cocytius* group, and the remaining Sphingini, in addition to Acherontiini. Relationships among these four potential tribes according to the molecular analysis are largely congruent with the morphological phylogeny of Kitching [33] if the trees in the latter are re-rooted at the *Xanthopan*/*Cocytius* group.

Basal divergences within “Sphingini *sensu stricto*” (node 18, Fig. 2) are resolved with very strong support. As currently sampled, this clade appears to have an Old World tropical origin, as the Afrotropical *Dovania* is strongly placed as sister group to the remaining genera. We predict that the other, currently unsampled Old World sphingine genera placed near *Dovania* by Kitching and Cadiou [2] (e.g., the Afrotropical *Hoplistopus*, *Litosphingia*, *Praedora*, and the Oriental *Apocalypsis* and *Pseudodolbina*), will also prove to fall near the base of this clade.

The remaining Sphingini *sensu stricto* (node 24, Fig. 2) appear to represent an ancestrally and predominantly New World radiation, with repeated invasion of the north temperate regions from the tropics in both main lineages (centered on *Manduca* and *Sphinx*). We predict that the latter pattern will become clearer with further sampling, as our current exemplar selection is biased towards the Nearctic. Our sampling of the large genera *Lintneria* and *Sphinx* provides a probable instance of this postulated latitudinal trend. The two Neotropical species of *Lintneria*, *L. istar* and *L. merops*, are strongly placed (node 102, BP = 100%) as the sister group of *Sphinx* (plus two smaller genera; node 38, BP = 100%). The latter are all north temperate. Until recently, *Lintneria* was treated as part of a larger concept of *Sphinx*, despite Forbes [94] separation of it on the basis of a unique mesothoracic dorsal hump in the larva. Recent larval rearings have now corroborated these earlier observations by showing that the first four instars of all members of *Lintneria* for which they are known have this large, fleshy, anteriorly angled, dorsal protuberance, and the adults also have unique wing markings that are not found in *Sphinx sensu stricto* [51]. Our results are consistent thus with the reinstatement of *Lintneria* [51]. Relationships within the north temperate clade of *Sphinx* are not strongly resolved, possibly reflecting a very recent radiation, but the conifer feeding genera *Isoparce* and *Lapara* are nested within *Sphinx* and their generic status may need to be reconsidered.

### Relationships within Macroglossinae

Basal divergences within Macroglossinae are less strongly resolved than those within its sister group Smerinthinae+Sphinginae, and correspond poorly to current classification, paralleling the lack of clear morphological divisions within this subfamily [2]. Three main lineages can be provisionally recognized. While only three samples could be included, there is very strong support for monophyly of Dilophonotini: Hemarina (node 80, BP = 100%, Fig. 3), as predicted by morphology [2]. The remaining macroglossines are grouped into two clades, each moderately well supported; these are placed as sister groups in our tree, to the exclusion of Hemarina, but with very weak support. These two clades correspond strikingly to geographic distribution, but less so to current tribal/subtribal boundaries. One (node 19, BP = 68%), restricted entirely to the New World, which we tentatively call, “Dilophonotina *sensu lato*”, consists of Dilophonotina, Philampelini, and the *Proserpinus* group and a set of genera, typified by *Amphion* and *Sphecodina*, both currently placed in Macroglossini: Macroglossina. Its ostensible sister group (node 8, BP = 72%), consisting of all remaining Macroglossina as well as Choerocampina, is ancestrally and predominantly confined to the Old World.

Genera within the New World clade fall into two main groups. One is a very strongly supported subgroup of dilophonotines (node 20, BP = 100%) that we term “Dilophonotina *sensu stricto*”, for which there are several synapomorphies in the male genitalia [2]. These sphingids, like Hemarina and many Old World Macroglossina, feed most commonly on euasterids, particularly Rubiaceae and/or Apocynaceae, and/or on other plants sharing with Apocynaceae the possession of latex or resin canals [95]. *Cautethia spuria*, whose placement within the New World clade is not strongly resolved, also feeds on Rubiaceae [90].

The other main New World lineage, moderately supported (node 28, BP = 72%), consists of additional Dilophonotina, Philampelini (represented here only by *Eumorpha*, as the Hawaiian endemic *Tinostoma* was not available for study), and most New World genera of Macroglossina. This grouping is a novel hypothesis, and no morphological synapomorphies are yet apparent, although a close relationship among some of the included Macroglossina had been tentatively postulated on the basis of their shared spinose tibiae [1], [2]. Its constituent species, however, differ strikingly from “Dilophonotina *sensu stricto*” and most Old World Macroglossina in host plant use, being almost entirely restricted to a recognized syndrome of sphingid host plant families [95] that includes Actinidiaceae, Dilleniaceae, Onagraceae and Vitaceae, all which are thought to share the presence of raphide crystals.

Within Macroglossini, there is strong support for inclusion of Choerocampina in a derived subset of Macroglossina; the two closest relatives in our sample are the Southeast Asian *Gnathothlibus* and *Eupanacra* (nodes 21, 27, BP≥95%). Monophyly of Choerocampina, strongly corroborated here (node 29, BP = 87%), is supported by a unique bat-detecting hearing organ formed from the swollen, scale-less, air-filled second labial palp segment and differentiated pilifer [2], [96]. Rothschild and Jordan [1] recognized three groups within Choerocampina, based largely on variations in the labial palp-pilifer hearing organ: (1) *Chaerocina* and *Euchloron*; (2) *Deilephila*, *Hyles*, *Rhodafra*, *Xylophanes*; (3) *Basiothia*, *Cechenena*, *Centroctena*, *Hippotion*, *Pergesa*, *Rhagastis*, and *Theretra*. Relationships among genera in our analysis, on the whole are strongly resolved, agree partially with this postulate. Three of the members of group 2, *Hyles*, *Rhodafra*, and *Xylophanes*, are indeed closely related, but the smallest clade including them also contains *Chaerocina*, and *Deilephila* is placed with group 3 rather than here. *Euchloron* is the sister taxon to group 2 thus re-defined (node 42, BP = 69%), rather than to *Chaerocina* alone, rendering group 1 polyphyletic. Members of group 3 are indeed phylogenetically contiguous, but the group is basal and paraphyletic with respect to group 2. Rothschild and Jordan [1] also postulated a sister group relationship between *Cechenena* and *Rhagastis*, which is very strongly supported in the present analysis (node 87, BP = 100%).

If we provisionally accept the monophyly of the three main lineages of Macroglossinae identified here, substantial changes to the current classification will be required to achieve a set of formally named monophyletic tribes and subtribes. As currently constituted, Dilophonotini and Macroglossini are both polyphyletic. If we further accept the weak to modest support for nesting of both Philampelini and part of Macroglossina within Dilophonotina (the strongest being node 28, BP = 72%), then Dilophonotina is also polyphyletic. One implication of this finding is that phylogenetic evidence for retaining Philampelini at tribal rank with its current composition is weak. Macroglossini can be restored to monophyly (“Macroglossini *sensu stricto*,” node 8) if the New World genera grouping with Dilophonotina are removed. Subtribe definition therein will be problematic, however, as Choerocampina are nested deep within Macroglossina.

Basal relationships within Macroglossinae were the main point of difference between our ML and MP results. In the MP strict consensus (Fig. S1), Hemarina do not lie at the base of Macroglossinae; rather, they fall at the base of Old World Macroglossina, as sister group to *Neogurelca*+*Sphingonaepiopsis*. The conflict cannot be called strong, as basal divergences are resolved even less strongly under MP than under ML. Inspection of the ML phylogram, however, suggested that the discrepancy might reflect long-branch attraction under MP [97], as both Hemarina and *Neogurelca*+*Sphingonaepiopsis* appear to be long-branched (Fig. S2). To explore this possibility further, the MP analysis was repeated with each group excluded in turn. When *Neogurelca* and *Sphingonaepiopsis* were excluded, Hemarina moved to base of the Macroglossinae as in the ML tree, whereas when Hemarina were excluded, *Neogurelca*+*Sphingonaepiopsis* moved to base of Sphingidae, allied with the longest branches in the analysis, namely those leading to the outgroups. These results are at least consistent with long-branch attraction.

### Biogeography

A notable finding of this study is that broad-scale geographic distribution in sphingids is more phylogenetically conserved than previous classifications had implied. The ancestral distribution for Smerinthinae+Sphinginae is very clearly Old World, while the great majority of Macroglossinae are divided into two probable sister groups with sharply contrasting Old World (Macroglossini *sensu stricto*; node 8, Fig. 3) versus New World (Dilophonotina *sensu lato*; node 19, Fig. 3) distributions. The most notable uncertainty is the ancestral distribution for the basal macroglossine lineage, Hemarina, which contains only *Hemaris* and *Cephonodes*. This ambiguity is probably the result of our limited sampling of *Hemaris*. The single species included here is atypical in being North American; most of the genus, like *Cephonodes*, is restricted to the Old World. We predict that further phylogenetic study of *Hemaris* will show this genus to be ancestrally Old World, thereby more securely establishing an Old World origin for Hemarina, Macroglossinae and Sphingidae as a whole. Appeal to related families, in contrast is unlikely to further clarify the issue. Molecular studies now place Sphingidae in a clade with Saturniidae and Bombycidae *sensu stricto* [31], [98]. Saturniidae appear to have a New World origin, and the Bombycidae divide basally into New World versus Old World sister groups [53].

## Supporting Information

Table S1 The sampled 131 ingroup and 10 outgroup taxa with specimen localities, LepTree voucher identification numbers, and GenBank accession numbers.
(0.29 MB DOC)

Figure S1 Strict consensus of the 12 MPCs (length = 42618 steps, CI = 0.15, RI = 0.53) resulting from five-gene simultaneous MP analysis. Nodes are labeled to the right of each internal branch. Bootstrap values below branches, Bremer supports above.
(2.03 MB TIF)

Figure S2 ML phylogram. lnL = −187418.656372. The scale bar indicates the estimated substitutions per site.
(1.73 MB TIF)

Dataset S1 Data matrix. The aligned sequence data are presented in sequential Nexus format.
(0.96 MB DOC)
