# Supplementary material for: Phylogeny and Biogeography of Hawkmoths (Lepidoptera: Sphingidae): Evidence from Five Nuclear Genes
Source: PLoS One. 2009 May 28;4(5):e5719. doi: 10.1371/journal.pone.0005719 (PMC2683934; doi:10.1371/journal.pone.0005719)
Supplement: Table S1 — The sampled 131 ingroup and 10 outgroup taxa with specimen localities, LepTree voucher identification numbers, and GenBank accession numbers. (0.29 MB DOC) [file pone.0005719.s001.doc]

**Table S1** The sampled 131 ingroup and 10 outgroup taxa with specimen localities, LepTree identification numbers, and GenBank accession numbers.

| **Ingroup taxa** | **Locality** | **LepTree ID No.** | ***CAD*** | ***DDC*** | ***EF-1α*** | ***PER*** | ***WG*** |
| --- | --- | --- | --- | --- | --- | --- | --- |
| **Sphinginae Latreille, [1802]** |  |  |  |  |  |  |  |
| **Sphingini Latreille, [1802]** |  |  |  |  |  |  |  |
| *Ceratomia catalpae* (Boisduval, [1875]) | USA: Arkansas | DCR-02-1870 | EU479036 | EU479146 | EU479256 | EU479366 | EU479470 |
| *Ceratomia amyntor* (Geyer, [1835]) | USA: Colorado | RSP-96-0932 | - | AF234584 | AF234562 | - | - |
| *Cocytius duponchel* (Poey, 1832) | Costa Rica: Guanacaste | DHJ-02-2359 | EU479040 | EU479151 | EU479261 | EU479371 | EU479475 |
| *Dolba hyloeus* (Drury, 1773) | USA: Texas | RSP-02-1575 | EU479048 | EU479158 | EU479269 | EU479377 | EU479483 |
| *Dovania poecila* Rothschild & Jordan, 1903 | Kenya: Kakamega | IJK-03-3169 | EU479050 | - | - | EU479379 | EU479485 |
| *Euryglottis dognini* Rothschild, 1896 | Ecuador: Morona-Santiago | WJK-03-2891 | EU479060 | EU479170 | EU479281 | EU479387 | EU479495 |
| *Isoparce cupressi* (Boisduval, [1875]) | USA: North Carolina | JBS-05-0001 | EU479066 | EU479178 | EU479290 | EU479393 | EU479502 |
| *Lapara coniferarum* (Smith, 1797) | USA: Georgia | JKA-02-1670 | EU032697 | EU032833  EU032834 | AF234569 | EU032997 | EU033100 |
| *Lintneria istar* (Rothschild & Jordan, 1903) | USA: Texas | CWB-02-1591 | EU479110 | EU479222 | EU479335 | EU479435 | EU479548 |
| *Lintneria merops* (Boisduval, 1870) | Ecuador: Manabi | WJK-03-2198 | EU479112 | EU479224 | EU479337 | EU479437 | EU479550 |
| *Macropoliana natalensis* (Butler, 1875) | USA: Arizona | IJK-03-3211 | EU479073 | EU479184 | - | EU479399 | EU479509 |
| *Manduca florestan* (Stoll, 1782) | Tanzania: Usumbara Mts. | JPT-02-1529 | - | EU479186 | EU479298 | - | EU479511 |
| *Manduca muscosa* (Rothschild & Jordan, 1903) | USA: Arizona | JBW-02-1508 | - | EU479187 | EU479299 | - | EU479512 |
| *Manduca quinquemaculatus* (Haworth, 1803) | USA: Arkansas | DCR-02-1876 | EU479075 | EU479188 | EU479300 | EU479401 | EU479513 |
| *Manduca sexta* (Linnaeus, 1763) | Argentina: Córdoba | MM-03-2154 | EU032707 | EU032841 | AF234571 | EU033005 | EU033109 |
| *Meganoton analis* (Felder, [1874]) | Japan: Shizuoka | AYK-04-2579 | EU479078 | EU479191 | EU479303 | EU479404 | EU479516 |
| *Neococytius cluentius* (Cramer, 1775) | Ecuador: Pichincha | WJK-03-1949 | EU479081 | EU479194 | EU479306 | EU479407 | EU479519 |
| *Paratrea plebeja* (Fabricius, 1777) | USA: Georgia | WJK-02-1939 | EU479091 | EU479203 | EU479316 | EU479417 | EU479529 |
| *Psilogramma increta* (Walker, [1865]) | Philippines: Palawan | IJK-02-5988 | EU479102 | EU479214 | EU479327 | EU479428 | EU479540 |
| *Sphinx caligineus* (Butler, 1877) | China: Beijing | IJK-04-0012 | EU479108 | EU479220 | EU479333 | EU479433 | EU479546 |
| *Sphinx chersis* (Hübner, [1823]) | USA: Arizona | JPT-xx-0839 | - | AF234596 | AF234577 | - | - |
| *Sphinx dollii* Neumoegen, 1881 | USA: Arizona | JPT-02-1532 | EU479109 | EU479221 | EU479334 | EU479434 | EU479547 |
| *Sphinx kalmiae* Smith, 1797 | USA: Georgia | WJK-02-1938 | EU479111 | EU479223 | EU479336 | EU479436 | EU479549 |
| *Xanthopan morganii* (Walker, 1856) | Tanzania: Usumbara Mts, | IJK-03-3209 | EU479118 | - | - | EU479442 | EU479556 |
|  |  |  |  |  |  |  |  |
| **Acherontiini Boisduval, [1875]** |  |  |  |  |  |  |  |
| *Acherontia styx* Westwood, 1847 | Philippines: Palawan | IJK-02-5989 | EU479012 | EU479122 | EU479232 | EU479346 | EU479446 |
| *Agrius cingulata* (Fabricius, 1775) | USA: Georgia | WJK-02-1941 | EU479018 | EU479128 | EU479238 | EU479352 | EU479452 |
| *Coelonia fulvinotata* (Butler, 1875) | Zimbabwe: bred stock | IJK-02-5816 | EU479041 | EU479152 | EU479262 | - | EU479476 |
| *Megacorma obliqua* (Walker, 1856) | Malaysia: Perak | AYK-04-0154 | EU479077 | EU479190 | EU479302 | EU479403 | EU479515 |
|  |  |  |  |  |  |  |  |
| **Smerinthinae Grote & Robinson, 1865** |  |  |  |  |  |  |  |
| **Smerinthini Grote & Robinson, 1865** |  |  |  |  |  |  |  |
| *Afroclanis calcareus* ( Rothschild & Jordan, 1907) | Tanzania: Udzungua | MF-05-0005 | EU479017 | EU479127 | EU479237 | EU479351 | EU479451 |
| *Amorpha juglandis* (Smith, 1797) | USA: Texas | CWB-02-1595 | EU479021 | EU479131 | EU479241 | EU479354 | EU479455 |
| *Andriasa contraria* Walker, 1856 | Kenya: Kakamega | IJK-03-3153 | EU479026 | - | - | EU479357 | EU479461 |
| *Callambulyx tatarinovii* (Bremer & Grey, 1853) | Japan: Shizuoka | AYK-04-2581 | EU479030 | EU479140 | EU479250 | EU479361 | - |
| *Chloroclanis virescens* (Butler, 1882) | Kenya: Kakamega | IJK-03-3160 | EU479037 | EU479148 | EU479258 | EU479368 | EU479472 |
| *Clanis bilineata* (Walker, 1866) | Taiwan: Nantou | AYK-04-0184 | EU479038 | EU479149 | EU479259 | EU479369 | EU479473 |
| *Cypa decolor* (Walker, 1856) | Malaysia: Pahang | AYK-04-0110 | EU479042 | EU479153 | EU479263 | EU479372 | EU479477 |
| *Daphnusa ocellaris* Walker, 1856 | Malaysia: Pahang | AYK-04-0240 | EU479044 | - | EU479265 | - | EU479479 |
| *Langia zenzeroides* Moore, 1872 | Japan: Nagano | AYK-04-2580 | EU479069 | EU479180 | EU479293 | EU479395 | EU479505 |
| *Laothoe populi* (Linnaeus, 1758) | United Kingdom: London | IJK-02-0012 | EU479070 | EU479181 | EU479294 | EU479396 | EU479506 |
| *Likoma apicalis Rothschild* & Jordan, 1903 | Tanzania: Mbega | MF-05-0008 | EU479071 | EU479182 | EU479295 | EU479397 | EU479507 |
| *Marumba quercus* ([Denis & Schiffermuller], 1775) | France: nr. Toulon | IJK-02-0118 | EU479076 | EU479189 | EU479301 | EU479402 | EU479514 |
| *Mimas tiliae* (Linnaeus, 1758) | United Kingdom: London | IJK-02-5836 | EU479079 | EU479192 | EU479304 | EU479405 | EU479517 |
| *Neoclanis basalis* (Walker, 1866) | Tanzania: Morogoro | IJK-03-3225 | EU479080 | EU479193 | EU479305 | EU479406 | EU479518 |
| *Neopolyptychus compar* (Rothschild & Jordan, 1903) | Tanzania: Usumbara Mts. | IJK-03-3207 | EU479083 | EU479195 | EU479308 | EU479409 | EU479521 |
| *Pachysphinx modesta* (Harris, 1839) | USA: Colorado | RSP-96-0626 | - | - | AF234573 | - | - |
| *Pachysphinx occidentalis* (Edwards, 1875) | USA: Arizona | JPT-02-1528 | EU479090 | EU479201 | EU479315 | EU479416 | EU479528 |
| *Paonias excaecata* (Smith, 1797) | USA: West Virginia | CWM-96-0573 | - | - | AF234572 | - | - |
| *Paonias myops* (Smith, 1797) | USA: Arizona  USA: Michigan | JPT-02-1540 MCN-03-1796 | EU032735 | EU479202 | AF234574 | EU033024 | EU033131 |
| *Parum colligata* (Walker, 1856) | Taiwan: Nantou | AYK-04-0182 | EU479092 | EU479204 | EU479317 | EU479418 | EU479530 |
| *Phyllosphingia dissimilis* (Bremer, 1861) | Taiwan: Nantou | AYK-04-0176 | EU479095 | EU479207 | EU479320 | EU479421 | EU479533 |
| *Polyptychoides digitatus* (Karsch, 1891) | Kenya: Kakamega | IJK-03-3159 | EU479097 | EU479209 | EU479322 | EU479423 | EU479535 |
| *Polyptychus andosa* (Walker, 1856) | Tanzania: Usumbara Mts. | IJK-03-3199 | EU479096 | EU479208 | EU479321 | EU479422 | EU479534 |
| *Pseudoclanis kenyae* Clark, 1928 | South Africa: bred stock | IJK-02-5839 | EU479100 | EU479212 | EU479325 | EU479426 | EU479538 |
| *Smerinthus cerisyi* Kirby, 1837 | USA: Wisconsin | JJK-95-0827 | - | AF234595 | AF234576 | - | - |
| *Smerinthus saliceti* (Boisduval, [1875] | USA: Arizona | JBW-02-1511 | EU479105 | EU479217 | EU479330 | EU479430 | EU479543 |
| *Viriclanis kingstoni* Aarvik, 1999 | Tanzania: Uzungura | MF-05-0010 | EU479117 | EU479228 | EU479342 | - | EU479555 |
|  |  |  |  |  |  |  |  |
| **Sphingulini Rothschild & Jordan, 1903** |  |  |  |  |  |  |  |
| *Dolbina tancrei* Staudinger, 1887 | Japan: Yamanashi | AYK-04-2557 | EU479049 | EU479159 | EU479270 | EU479378 | EU479484 |
| *Hopliocnema brachycera* (Lower, 1897) | Australia: Northern Territory | MJM-96-0232 | EU032684 | EU032817 | EU479286 | EU032984 | EU033089 |
| *Kentrochrysalis consimilis* Rothschild & Jordan, 1903 | Japan: Yamanashi | AYK-04-2571 | EU479067 | EU479179 | EU479291 | EU479394 | EU479503 |
|  |  |  |  |  |  |  |  |
| **Ambulycini Butler, 1876** |  |  |  |  |  |  |  |
| *Adhemarius daphne* (Boisduval, 1875) | Mexico: San Luis Potosi | JKA-02-1642 | EU479015 | EU479125 | EU479235 | EU479349 | EU479449 |
| *Ambulyx schauffelbergeri* Bremer & Grey, 1853 | Japan: Yamanashi | AYK-04-2568 | EU479020 | EU479130 | EU479240 | - | EU479454 |
| *Amplypterus mansoni* (Clark, 1924) | Taiwan: Nantou | AYK-04-0174 | - | EU479135 | EU479245 | - | EU479459 |
| *Amplypterus panopus* (Cramer, 1779) | Malaysia: Pahang | AYK-04-0276 | EU479025 | EU479136 | EU479246 | EU479356 | EU479460 |
| *Protambulyx euryalus* Rothschild & Jordan, 1903 | Ecuador: Morona-Santiago | WJK-03-1945 | EU479099 | EU479211 | EU479324 | EU479425 | EU479537 |
|  |  |  |  |  |  |  |  |
| **Macroglossinae Harris, 1839** |  |  |  |  |  |  |  |
| **Macroglossini Harris, 1839** |  |  |  |  |  |  |  |
| **Macroglossina Harris, 1839** |  |  |  |  |  |  |  |
| *Acosmerycoides harterti* (Rothschild, 1895) | Malaysia: Pahang | AYK-04-0200 | EU479013 | EU479123 | EU479233 | EU479347 | EU479447 |
| *Acosmeryx naga* (Moore, [1858]) | Japan: Yamanashi | AYK-04-2570 | EU479014 | EU479124 | EU479234 | EU479348 | EU479448 |
| *Ampeophaga dolichoides* (Felder, [1874]) | Malaysia: Pahang | AYK-04-0121 | EU479022 | EU479132 | EU479242 | - | EU479456 |
| *Ampelophaga rubiginosa* Bremer & Grey, 1853 | Japan: Yamanashi | AYK-04-2573 | EU479023 | EU479133 | EU479243 | EU479355 | EU479457 |
| *Amphion floridensis* Clark, 1920 | USA: Florida | JYM-05-0003 | EU479024 | EU479134 | EU479244 | - | EU479458 |
| *Angonyx testacea* (Walker, 1856) | Taiwan: Pingtung | AYK-04-0243 | EU479027 | EU479137 | EU479247 | EU479358 | EU479462 |
| *Clarina kotschyi* (Kollar, [1849]) | Turkey: Alanya | IJK-04-0005 | EU479039 | EU479150 | EU479260 | EU479370 | EU479474 |
| *Daphnis nerii* (Linnaeus, 1758) | France: bred stock | IJK-02-5810 | EU479043 | EU479154 | EU479264 | EU479373 | EU479478 |
| *Darapsa myron* (Cramer, 1779) | USA: bred stock | IJK-02-5963 | EU479045 | EU479155 | EU479266 | EU479374 | EU479480 |
| *Deidamia inscriptum* (Harris, 1839) | Canada: Toronto | AYK-04-5756 | EU479046 | EU479156 | EU479267 | EU479375 | EU479481 |
| *Elibia dolichus* (Westwood, 1847) | Malaysia: Pahang | AYK-04-0192 | EU479051 | EU479160 | EU479271 | EU479380 | EU479486 |
| *Enpinanga borneensis* (Butler, 1879) | Malaysia: Kuala Lipis | AYK-04-0104 | EU479052 | EU479161 | EU479272 | EU479381 | EU479487 |
| *Eupanacra regularis* (Butler, 1875) | Malaysia: Kuala Lipis | AYK-04-0103 | EU479057 | EU479167 | EU479278 | EU479384 | EU479492 |
| *Euproserpinus phaeton* Grote & Robinson 1865 | USA: California | DR-05-9000 | EU479058 | EU479168 | EU479279 | EU479385 | EU479493 |
| *Gnathothlibus erotus* (Cramer, 1777) | Malaysia: Pahang | AYK-04-0155 | EU479061 | EU479171 | EU479282 | EU479388 | EU479496 |
| *Macroglossum stellatarum* (Linnaeus, 1758) | France: bred stock | IJK-02-5806 | EU479072 | EU479183 | EU479296 | EU479398 | EU479508 |
| *Neogurelca himachala* (Butler, [1876]) | China: Zhejiang | IJK-04-0004 | EU479082 | - | EU479307 | EU479408 | EU479520 |
| *Nephele accentrifera* (Palisot de Beauvois, [1821]) | Kenya: Kakamega | IJK-03-3161 | EU479084 | - | EU479309 | EU479410 | EU479522 |
| *Proserpinus clarkiae* (Boisduval, 1852) | Unknown | - | - | - | AF173394 | - | - |
| *Proserpinus lucidus* (Boisduval, 1852) | USA: California | AYK-04-0065 | EU479028 | EU479138 | EU479248 | EU479359 | EU479463 |
| *Proserpinus terlooii* Edwards 1875 | USA: Arizona | JBW-02-1513 | EU479098 | EU479210 | EU479323 | EU479424 | EU479536 |
| *Sphecodina abbottii* (Swainson, 1821) | USA: Maryland | AYK-04-0366 | EU479106 | EU479218 | EU479331 | EU479431 | EU479544 |
| *Sphingonaepiopsis gorgoniades* (Hübner, [1819]) | Ukraine | IJK-05-0003 | EU479107 | EU479219 | EU479332 | EU479432 | EU479545 |
| *Temnora eranga* (Holland, 1889) | Kenya: Kakamega | IJK-03-3157 | EU479113 | - | EU479338 | EU479438 | EU479551 |
|  |  |  |  |  |  |  |  |
| **Choerocampina Grote & Robinson, 1865** |  |  |  |  |  |  |  |
| *Basiothia medea* (Fabricius, 1781) | Mauritius: Mare de Vacoas | IJK-02-0005 | EU479029 | EU479139 | EU479249 | EU479360 | EU479464 |
| *Cechenena helops* (Walker, 1856) | Malaysia: Pahang | AYK-04-0168 | EU479033 | EU479143 | EU479253 | EU479364 | EU479467 |
| *Cechenena subangustata* Rothschild, 1920 | Taiwan: Taiitung | AYK-04-0214 | EU479034 | EU479144 | EU479254 | - | EU479468 |
| *Chaerocina dohertyi* Rothschild & Jordan, 1903 | Kenya: Kakamega | IJK-03-3166 | - | EU479147 | EU479257 | EU479367 | EU479471 |
| *Deilephila elpenor* (Linnaeus, 1758) | United Kingdom: Oxfordshire | IJK-02-5866 | EU479047 | EU479157 | EU479268 | EU479376 | EU479482 |
| *Euchloron megaera* (Linnaeus, 1758) | Kenya: Kakamega | IJK-03-3155 | EU479055 | EU479164 | EU479275 | - | EU479490 |
| *Hippotion celerio* (Linnaeus, 1758) | Czech Republic | IJK-02-5932 | EU479064 | EU479174 | EU479285 | EU479391 | - |
| *Hyles hippophaes* (Esper, 1789) | France: Hautes Alpes | IJK-02-5817 | - | EU479175 | EU479287 | - | EU479499 |
| *Hyles lineata* (Fabricius, 1775) | USA: Arkansas  USA: Colorado | DCR-02-1881 RSP-96-0929 | EU032688 | EU479176 | EU479288 | EU032988 | EU479500 |
| *Pergesa acteus* (Cramer, 1779) | Malaysia: Pahang  Taiwan: Hualien | AYK-04-0117 AYK-04-0231 | EU479093 | EU479205 | EU479318 | EU479419 | EU479531 |
| *Rhagastis mongoliana* (Butler, [1876]) | Japan: Shizuoka | AYK-04-2556 | EU479103 | EU479215 | EU479328 | EU479429 | EU479541 |
| *Rhodafra marshalli* Rothschild & Jordan, 1903 | Tanzania: Kifulo Plateau | MF-05-0009 | EU479104 | EU479216 | EU479329 | - | EU479542 |
| *Theretra alecto* (Linnaeus, 1758) | Cyprus | IJK-02-5880 | EU479114 | EU479225 | EU479339 | EU479439 | EU479552 |
| *Theretra capensis* (Linnaeus, 1764) | Tanzania: Pwani | IJK-03-3184 | EU479115 | EU479226 | EU479340 | EU479440 | EU479553 |
| *Xylophanes chiron* (Drury, 1773) | Costa Rica: Tabaean | IJK-02-5904 | EU479119 | EU479229 | EU479343 | EU479443 | - |
| *Xylophanes falco* (Walker, 1856) | USA: Arizona | JPT-xx-0838 | - |  |  | - | - |
| *Xylophanes porcus* (Hübner, [1823]) | Costa Rica: Guanacaste | DHJ-02-2369 | EU479120 | EU479230 | EU479344 | EU479444 | EU479557 |
| *Xylophanes tersa* (Linnaeus, 1771) | USA: Arkansas | DCR-02-1879 | EU479121 | EU479231 | EU479345 | EU479445 | EU479558 |
|  |  |  |  |  |  |  |  |
| **Dilophonotini Burmeister, 1878** |  |  |  |  |  |  |  |
| **Dilophonotina Burmeister, 1878** |  |  |  |  |  |  |  |
| *Aellopos ceculus* (Cramer, 1777) | Costa Rica: Guanacaste | DHJ-02-2399 | EU479016 | EU479126 | EU479236 | EU479350 | EU479450 |
| *Aellopos tantalus* (Linnaeus, 1758) | USA: Florida | RSP-95-1070 | - | AF234582 | AF234559 | - | - |
| *Aleuron choloroptera* (Perty, [1833]) | Belize: Cayo District | AYK-04-0506 | EU479019 | EU479129 | EU479239 | EU479353 | EU479453 |
| *Callionima falcifera* (Fabricius, 1775) | British Virgin Islands: Guana Island | RFD-96-0966 | EU479031 | EU479141 | EU479251 | EU479362 | EU479465 |
| *Cautethia spuria* (Boisduval, [1875]) | Mexico: San Luis Potosi | JKA-02-1668 | EU479032 | EU479142 | EU479252 | EU479363 | EU479466 |
| *Enyo ocypete* (Linnaeus, 1758) | Costa Rica: Guanacaste | DHJ-02-2390 | EU479053 | EU479162 | EU479273 | EU479382 | EU479488 |
| *Erinnyis ello* (Linnaeus, 1758) | USA: Arizona | JPT-02-1542 | EU479054 | EU479163 | EU479274 | EU479383 | EU479489 |
| *Eupyrrhoglossum sagra* (Poey, 1832) | Costa Rica: Guanacaste | DHJ-04-46898 | EU479059 | EU479169 | EU479280 | EU479386 | EU479494 |
| *Hemeroplanes ornatus* Rothschild, 1894 | Costa Rica: Heredia | AYK-04-0003 | EU479063 | EU479173 | EU479284 | EU479390 | EU479498 |
| *Isognathus rimosa* (Grote, 1865) | Mexico: El lobo  Dominican Republic: Pedernales | JKA-02-1646  AYK-04-0348 | EU479065 | EU479177 | EU479289 | EU479392 | EU479501 |
| *Kloneus babayaga* Skinner, 1923 | Costa Rica: Guanacaste | DHJ-04-2375 | EU479068 | - | EU479292 | - | EU479504 |
| *Madoryx plutonius* (Hübner, [1819]) | Costa Rica: Heredia | AYK-04-0029 | EU479074 | EU479185 | EU479297 | EU479400 | EU479510 |
| *Nyceryx magna* (Felder, [1874]) | Costa Rica: Guanacaste | DHJ-02-2378 | EU479085 | EU479196 | EU479310 | EU479411 | EU479523 |
| *Oryba kadeni* (Schaufuss, 1870) | Costa Rica: Guanacaste | DHJ-04-55866 | EU479086 | EU479197 | EU479311 | EU479412 | EU479524 |
| *Pachygonidia subhamata* (Walker, 1856) | Costa Rica: Guanacaste | DHJ-04-61221 | EU479087 | EU479198 | EU479312 | EU479413 | EU479525 |
| *Pachylia ficus* (Linnaeus, 1758) | Mexico: El Lobo | JKA-02-1644 | EU479088 | EU479199 | EU479313 | EU479414 | EU479526 |
| *Pachylioides resumens* (Walker, 1856) | Costa Rica: Heredia | AYK-04-0002 | EU479089 | EU479200 | EU479314 | EU479415 | EU479527 |
| *Perigonia ilus* Boisduval, 1870 | Ecuador: Manabí | WJK-03-2191 | EU479094 | EU479206 | EU479319 | EU479420 | EU479532 |
| *Pseudosphinx tetrio* (Linnaeus, 1771) | Dominican Republic: Puerto Escondido | AYK-04-0334 | EU479101 | EU479213 | EU479326 | EU479427 | EU479539 |
| *Unzela japix* (Cramer, 1776) | Costa Rica: Guanacaste | DHJ-02-2296  DHJ-02-2376 | EU479116 | EU479227 | EU479341 | EU479441 | EU479554 |
|  |  |  |  |  |  |  |  |
| **Hemarina Tutt, 1902** |  |  |  |  |  |  |  |
| *Cephonodes hylas* (Linnaeus, 1771) | Unknown | IJK-02-5931 | EU479035 | EU479145 | EU479255 | EU479365 | EU479469 |
| *Hemaris diffinis* (Boisduval, 1836) | USA: Arkansas | DCR-02-1882 | EU479062 | EU479172 | EU479283 | EU479389 | EU479497 |
| *Hemaris diffinis* (Boisduval, 1836) | USA: Maryland | TPF-94-1450 | EU032692 | EU032826  EU032827 | AF234568 | - | EU033096 |
|  |  |  |  |  |  |  |  |
| **Philampelini Burmeister, 1878** |  |  |  |  |  |  |  |
| *Eumorpha achemon* (Drury, 1773) | USA: Arizona | JPT-02-1533 | EU479056 | EU479165 | EU479276 | - | EU479491 |
| *Eumorpha pandorus* (Hübner, [1821]) | USA: Indiana | CWM-95-0830 | - | AF234588 | AF234565 | - | - |
| *Eumorpha typhon* (Klug, 1836) | USA: Arizona | JBW-02-1504 | - | EU479166 | EU479277 | - | - |
|  |  |  |  |  |  |  |  |
| **Outgroup taxa** |  |  |  |  |  |  |  |
| *Acanthobrahmaea europaea* (Hartig, 1963) | Italy | RSP-95-0990 | EU032638 | EU032773 | AF234558 | EU032950 | EU033053 |
| *Apha aequalis* (Felder, 1874) | Japan: Yamanashi | AYK-04-2506 | EU032628,  EU032629 | - | - | - | EU033047 |
| *Bombyx mori* (Linnaeus, 1758) | Lab colony | UNK-90-0062 | EU032656 | EU032793 | EU490615 | EU032965 | EU033069 |
| *Endromis versicolora* (Linnaeus, 1758) | Germany, Roding | RSP-95-1108 | EU032680 | EU032813 | AF234566 | EU032980 | EU033085 |
| *Janiodes sp.* | Ecuador: Morona Santiago | KLW-03-2869 | EU032694 | EU032829 | EU490616 | EU032994 | EU033097 |
| *Lemonia dumi* (Linnaeus, 1761) | Sweden: Svartbacken | IJK-94-0551 | EU032699 | EU032836 | AF234570 | EU032999  EU033000 | EU033102 |
| *Macrothylacia rubi* (Linnaeus, 1758) | Spain, Barcelona | RSP-xx-0840 | EU032708 | EU032842 | AF234553 | EU033006 | EU033110 |
| *Mirina christophi* (Staudinger, 1887) | South Korea: Taeweon SA | RXR-02-0511 | EU032703 | - | - | - | EU033106 |
| *Oberthueria formosibia* (Matsumura, 1927) | Taiwan: Taitung | AYK-04-0824 | EU032717,  EU032718 | - | - | - | EU033115 |
| *Phiditia sp.* | Brazil: Amazonas | RWH-96-0916 | EU032726 | EU032856 | EU490617 | - | EU033123 |
