# Supplementary figures and images for: Phylogeny and Biogeography of Hawkmoths (Lepidoptera: Sphingidae): Evidence from Five Nuclear Genes
Source: PLoS One. 2009 May 28;4(5):e5719. doi: 10.1371/journal.pone.0005719 (PMC2683934; doi:10.1371/journal.pone.0005719)

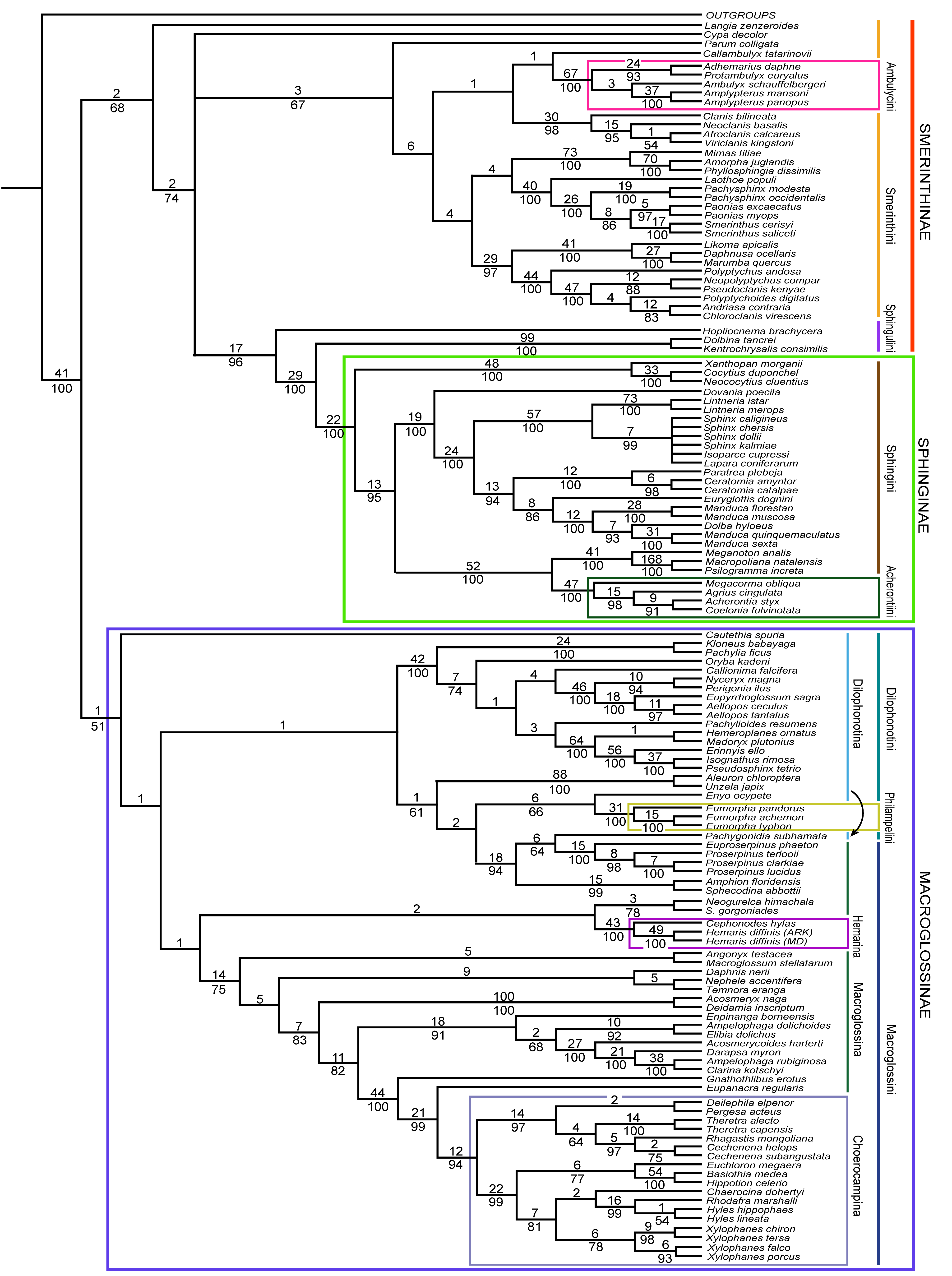

Supplement: Figure S1 — Strict consensus of the 12 MPCs (length = 42618 steps, CI = 0.15, RI = 0.53) resulting from five-gene simultaneous MP analysis. Nodes are labeled to the right of each internal branch. Bootstrap values below branches, Bremer supports above. (2.03 MB TIF) [file pone.0005719.s002.tif]

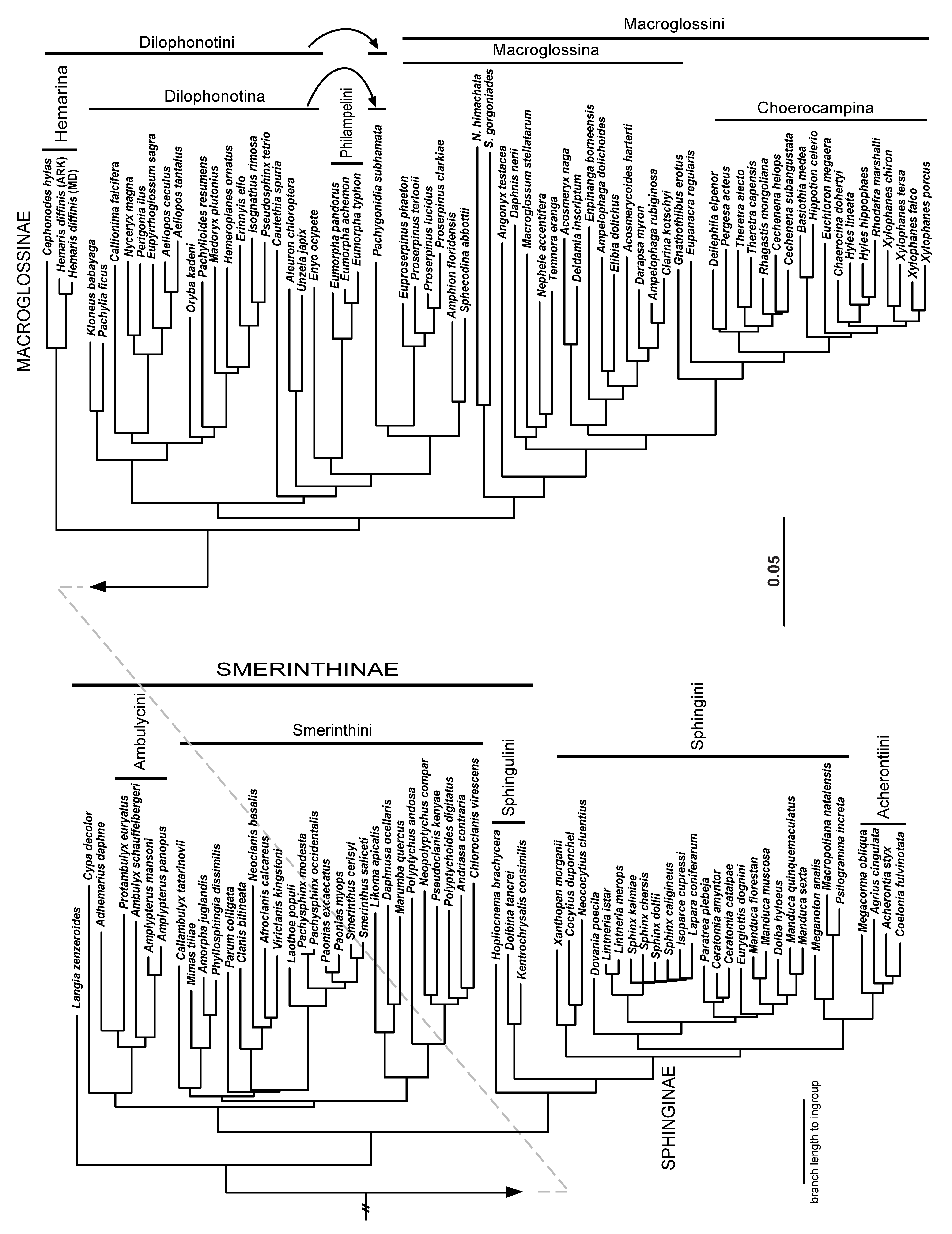

Supplement: Figure S2 — ML phylogram. lnL = −187418.656372. The scale bar indicates the estimated substitutions per site. (1.73 MB TIF) [file pone.0005719.s003.tif]
